# Effects of Aqueous and Methanolic Extracts of Stem Bark of *Alstonia boonei* De Wild. (Apocynaceae) on Dextran Sodium Sulfate-Induced Ulcerative Colitis in Wistar Rats

**DOI:** 10.1155/2020/4918453

**Published:** 2020-05-30

**Authors:** Carine Flore Adjouzem, Ateufack Gilbert, Marius Mbiantcha, William Yousseu Nana, Vanessa Matah Marthe Mba, Stephanie Flore Djuichou Nguemnang, Eric Gonzal Tsafack, Albert Donatien Atsamo

**Affiliations:** ^1^Laboratory of Animal Physiology and Phytopharmacology, Department of Animal Biology, Faculty of Science, University of Dschang, P.O. Box 67, Dschang, Cameroon; ^2^Laboratory of Animal Physiology, Faculty of Science, University of Yaoundé I, P.O. Box 812, Yaoundé, Cameroon

## Abstract

Among the most exploited species in Cameroon, *Alstonia boonei* is widely used in African medicine for the relief of several pathologies including gastrointestinal disorders. This study was conducted in order to assess the effects of aqueous and methanol stem-bark extracts of *Alstonia boonei* on DSS- (dextran sodium sulfate-) induced intestinal colitis and to determine its antioxidant potential. The classes of secondary metabolites present in these extracts were determined by chemical screening. The production of TNF-*α*, IL-6, IL-1*β*, and PGE2 was performed by *in vitro* ELISA analysis. Anticolitis effects were determined using an *in vivo* model of ulcerative colitis induced by DSS. The colitis was induced with a double dose of DSS (3% and 1%), and the aqueous and methanol extracts were administered orally from the 6^th^ day after commencement of induction. The phytochemical screening revealed the presence of six classes of secondary metabolites in these crude extracts: tannins, saponins, alkaloids, steroids, flavonoids, and phenols. Methanol and aqueous extracts of *Alstonia boonei* significantly (*P* < 0.001) inhibited TNF-*α*, IL-6, IL-1*β*, and PGE2 production stimulated by LPS. Both extracts at all doses significantly reduced (*P* < 0.01, *P* < 0.001) the signs of DSS-induced colitis in the Wistar rats by decreasing inflammation and chronic colon damage. In addition, the extracts significantly (*P* < 0.001) reduced malondialdehyde and nitric oxide levels in the colon and significantly (*P* < 0.01) increased superoxide dismutase and catalase and reduced glutathione (*P* < 0.05). Both extracts showed greater activity than the reference substance (prednisolone 4 mg/kg) used in this study. This study has demonstrated that aqueous and methanol extracts of *Alstonia boonei* stem bark have healing properties against colitis experimentally induced by DSS in rats.

## 1. Introduction

Ulcerative colitis (UC), which is essentially confined to the colon and rectum, is an inflammatory bowel disease that causes noncontrolled inflammation of the bowel with disruption of the gastrointestinal tract [1–3]. In sufferers, this pathology is characterized by the appearance of symptoms such as diarrhea, weight loss, abdominal cramps, and mucous ulcers causing a recurrence of blood in the stool [4]. Genetic susceptibility, imbalance between the immune 1 system and gut microbes, the environment, and/or microbes are important factors that justify its pathogenicity [5–7]. In addition, several proinflammatory mediators like cytokines, eicosanoids, and ROS (reactive oxygen species) [8] play a major role in its development and its evolution. According to Lih-Brody et al. [9], ulcerative colitis is a disease usually related to a decrease in the antioxidant capacity of free radicals (ROS); in fact, an uncontrolled increase in ROS can cause lipid peroxidation, which results in an inhibition of the antioxidant capacity of cells leading to the development of significant colic inflammation [10]. Furthermore, it is known that people with colitis develop significant lipid peroxidation of membranes and disruption of DNA and tissue proteins as a result of exaggerated ROS production [11, 12]. The production of ROS by the body is under the control of a system of enzymatic antioxidant defenses consisting of GPx (glutathione peroxidase), SOD (superoxide dismutase), and CAT (catalase) and a nonenzymatic antioxidant defense system consisting of glutathione (GSH), nitric oxide (NO), malondialdehyde (MDA), and ascorbic acid. During the development of ulcerative colitis, symptoms resulting from an aggravation of the inflammatory process appear, which will result in an exaggerated production of ROS and many cytokines (proinflammatory) such as TNF-alpha, IL-6, and IL-1 beta [13]. Since high levels of cytokines (proinflammatory), like those of ROS, play a key role in the evolution and aggravation of inflammatory bowel diseases, it is clearly established clinically that molecules capable of inhibiting the production and/or activity of these proinflammatory mediators, as well as those capable of improving lipid peroxidation and/or trapping free radicals, are good candidates for the treatment of inflammatory bowel diseases [14, 15].

For maintaining long-term relief and controlling symptoms (bleeding, diarrhea, and abdominal pain) as well as decreasing colonic inflammation in people with ulcerative colitis, the recommended treatments nowadays are corticosteroids/immunosuppressants, noninflammatory anti-TNF-alpha, aminosalicylate, and immunodyes [16–18]. Nevertheless, in the vast majority of cases, these drugs are very inefficient and/or most of the time cause unbearable side effects such as hyperglycemia, muscle weakness, glaucoma, and/or even malignant tumors [17, 19, 20]. In order to effectively relieve people with ulcerative colitis, it is urgent to develop and implement new therapeutic substances effective for the treatment of this pathology [21], especially with very little or no toxic effect. In this context, the alternative treatment with medicinal plants offers encouraging options.

Africa has enormous biodiversity resources and it is estimated that it contains between 40,000 and 45,000 species of plants with development potential, of which 5,000 species are used for medicinal purposes [22]. Indeed, medicinal plants are the most easily accessible health resource for the community in many regions of Africa, and they most often represent the preferred option for the populations. The scientific literature has an increasing number of publications aimed at evaluating the efficacy of African medicinal plants which are supposed to make an important contribution to the maintenance of health and the introduction of new treatments [22]. Moreover several plant species have already shown their effectiveness against intestinal inflammation pathologies. This is how Mehrabani et al. [23] showed the effects of *Calendula officinalis* on acetic acid-induced ulcerative colitis in dogs as an animal model; Tanideh et al. [24] showed the healing effects of *Hypericum perforatum* extract on acetic acid-induced ulcerative colitis in rats; Rajendiran et al. [25] showed anti-inflammatory activity of *Alpinia officinarum* Hance on rat-colon inflammation and tissue damage in DSS-induced acute and chronic colitis models; and Shin et al. [26] showed that *Boehmeria nivea* attenuates the development of dextran sulfate sodium-induced experimental colitis.

*Alstonia boonei,* a member of the large family Apocynaceae (approximately 60 species), is a large deciduous tree with an average size of 45 meters and a diameter of about 1.2 meters [27]. This tree is widely distributed in Africa: Central African Republic, Egypt, Ghana, Ivory Coast, Nigeria, and Cameroon [28]. In the African pharmacopoeia, the roots, barks, and/or leaves of this tree are very widely used for primary healthcare of the populations. Thus, barks are used to relieve pain and to treat fever, chronic diarrhea, and rheumatoid arthritis, besides its use as antivenom for snake bites [29]. In addition, *A. boonei* has been reported in several scientific studies, and it appears that the extracts of this plant have antioxidant [30], antimicrobial [31], anti-inflammatory, antipyretic, analgesic, and antimalarial [32, 33] properties. Similarly, several phytochemical studies have shown that this plant is rich in alkaloids, saponins, flavonoids, terpenoids, steroids, tannins, and cardiac glycosides [30, 31, 34]. In addition compounds such as echitamine, N*α*-formylechitamidine, echitamidine, akuammidine, voacangine, N*α*-formyl-12-methoxyechitamidine, beta-amyrin, alpha-amyrin, ursolic acid, and lupeol have been isolated from foliage and/or bark of *A. boonei* [28]. Okoye et al. [35] showed the anti-inflammatory and antiulcer properties of beta-amyrin and/or alpha-amyrin isolated from *A. boonei*, while Obiagwu et al. [36] showed the in vitro antioxidant properties of the methanol extract of this plant. Other studies have shown that this plant was effective against lipid peroxidation [37] and was a nontoxic plant [37, 38]. In this study, we evaluated the effects of the aqueous and methanol extracts of the stem bark of *A. boonei* on the model of ulcerative colitis experimentally induced by DSS.

## 2. Materials and Methods

### 2.1. Reagents, Chemicals, and Equipment

The chemicals included methanol, dimethyl sulfoxide (DMSO), and sodium chloride purchased from Geochim Sarl, West Region of Cameroon. Dextran sodium sulfate was purchased from Tokyo Chemical Industry Co., LTD, Japan. Luminol, lymphocyte separation medium, lucigenin, and Hank's balanced salt solution were obtained from MP Biomedicals Inc., Research Organics, and Sigma. Phorbol myristate acetate and zymosan A were purchased from Fluka. Human monocytic leukemia cells were gotten from European Collection of Cell Cultures. Ammonium chloride of analytical grades and ethanol were from Merck Chemicals, Darmstadt, Germany. Human TNF-*α*, IL-1*β*, IL-6, and PGE2 ELISA Kit were from R&D Systems, Minneapolis, USA. Glass fiber filter and cell harvester were from Inotech, Dottikon, Switzerland. Prednisolone (Solupred) and pediatric catheter were purchased from local pharmacy.

### 2.2. Plant Material: Collection and Preparation

The stem bark of *A. boonei* was collected in Loum, littoral region of Cameroon, in March 2019. The plant species was identified by Mr. Nana Victor, a botanist at Cameroon National Herbarium in Yaoundé where our sample was authenticated by comparison with the available specimen having a voucher number 43368/HNC.

The barks were cleaned, cut into small pieces, dried under the shade, and crushed using a grinder to give a fine powder. The distilled water (4 L) was poured into a vessel previously containing 400 g of *A. boonei* powder, and then the mixture was boiled at 100°C for 25 min. After cooling, the mixture was filtered using a filter paper (No. 2) and dried in an oven set at 40°C, to obtain the crude extract (14.57 g, 3.7% extraction yield of aqueous extract). Another amount of *A. boonei* powder (300 g) was macerated in methanol (4.5 L) for 72 hours to give the methanol extract (16.18 g, 5.4% extraction yield) after filtration and removal of the solvent using a rotary evaporator.

### 2.3. Phytochemical Screening of *A. boonei*

The qualitative determination of triterpenes, steroids, phenols, tannins, flavonoids, saponins, alkaloids, and steroids in the aqueous and methanol extracts of *A. boonei* was carried out according to the standard protocols described by Matos [39].

## 3. *In Vitro* Assay

### 3.1. Assessment of Proinflammatory Cytokines and Prostaglandin E2

#### 3.1.1. Peritoneal Macrophages Isolation from Mice

Two NMRI (Naval Medical Research Institute) mice, weighing 24 and 26 grams each, received 1 mL (*i*.*p*.) of fetal bovine serum (FBS). Seventy-two (72) hours later, they were sacrificed by cervical dislocation. RPMI (10 mL, 10%) was injected into the peritoneum. Two minutes later, a 10 mL syringe was used to collect the contents of the peritoneal cavity which were immediately introduced into the tube and then centrifuged (20 min, 400 g, 4°C). After centrifugation, the supernatant was removed and 5 ml of incomplete RPMI added to the tube, followed by another centrifugation (10 min, 300 g, 4°C), after which 1 ml of the supernatant was once again removed and the incomplete RMPI diluted in HBSS. The trypan blue viability and hemocytometer count were assessed. The final concentration used for each test was 1 × 10^6^ cells/mL [40, 41].

#### 3.1.2. Isolation of Human Polymorphonuclear Neutrophils (PMNs)

Neutrophils were isolated by density gradient centrifugation (Ficoll-Hypaque). In fact, blood (10 mL) collected (aseptically) from a donor (33 years, voluntary and healthy) was immediately poured into a tube containing an anticoagulant (heparin), and then the solution of HBSS and the lymphocyte separation medium (LSM) were also introduced into the same tube at equal volume. After 30 minutes, the contents of the tube showed two layers; the upper layer was immediately removed and poured into another tube containing the LSM (5 mL) which was centrifuged (20 min, 400 g, room temperature). At the end of 20 minutes and after removal of the supernatant, distilled water (1 mL) was introduced into the tube, and after exactly 1 minute the HBSS (1 mL) was also introduced into the tube. Subsequently, 5 mL of HBSS was again introduced into the tube, and after centrifugation (10 min, 300 g, 4°C) and removal of the supernatant, 1 mL of HBSS was introduced into the tube which was stored in the ice [42]. Trypan blue viability and hemocytometer count were assessed. The final concentration used for each test was 1 × 10^6^ cells/mL.

The treatment of the animals was in agreement with the ICCBS Committee for the Protection, Use and Standardization of Animals (IACUC) (Protocol No. 1209004), and the study protocols were approved by the ICCB Ethics Committee, University of Karachi, Pakistan. The blood donor agreed on the use of his blood for the purpose of conducting this study.

#### 3.1.3. Chemoluminescence Assay

The aqueous or methanol extracts (25 *μ*l) of *A. boonei* or ibuprofen (3.125, 6.25, 12.5, 25, 50, and 100 *μ*g/mL) were introduced into wells (96-well white plate). Then diluted blood (1: 50 HBSS^++^), PMNs concentration of 1 × 10^6^ cell/mL, or macrophages of concentration 1 × 10^6^ cell/mL were also introduced into the wells; wells considered to be controls contained only HBSS^++^ and blood, PMNs, or macrophages. After incubation (luminometer chamber) of the plate for 20 minutes at 37°C, each well received 25 *μ*l of zymosan or PMA and/or 25 *μ*l of luminol or lucigenin of concentration 7 × 10^5^ M. Plaque yielded results as a relative light unit (RLU), and the percent inhibition was then calculated [40, 41].

(1) $$\begin{array}{c} \text{Inhibition}\left(\%\right)=\frac{\left({\text{RLU}}_{\text{control}}-{\text{RLU}}_{\text{sample}}\right)\times 100\;\%}{{\text{RLU}}_{\text{control}}}. \end{array}$$

#### 3.1.4. Cytokine and Prostaglandin E2 Assay

The two washed macrophages (PBS) (1 × 10^6^ cells/ml) were kept in culture in 5% CO_2_ (37°C, 24 h) and then in RPMI alone or mixed (2 *μ*g/ml of LPS), and then the extracts (2, 10, and 50 *μ*g/mL) were added, while the macrophages representing the controls were incubated only with the solvent. All the mixtures were then centrifuged (2500 g, 20 min), and then the supernatants were removed for the determination of TNF-*α*, IL- 6, IL-1*β*, and PGE2 using the ELISA kits obtained with the manufacturer's instructions [43].

#### 3.1.5. Cell Proliferation Test

For this test, a white round bottom plate (96 wells) and T lymphocytes concentrated at 2 × 20^6^ cells/ml were used. The test wells received the cells (50 *μ*L), 50 *μ*L of each extract or prednisolone diluted in RPMI (5%) at 2, 10, and 50 *μ*g/mL, and 50 *μ*L of phytohemagglutinin-L (PHA-L) of concentration 7.5 *μ*g/mL. The wells representing the negative control received 50 *μ*L of cells and 150 *μ*L of RPMI (5%), while the wells representing the positive control received the cells (50 *μ*L), 50 *μ*L of PHA, and 100 *μ*L of RPMI (5 %). The plate was then incubated (5% CO_2_, 72 h, 37°C); then each well received 25 *μ*L of concentrated (methyl-3H) thymidine at 0.5 *μ*Ci; after a second incubation (18 hours), cells were harvested using a glass filter (Inotech, Dottikon, Switzerland); and then counts per minute (liquid scintillation counter LS65000) were used to determine the level of thymidine infiltrated into the cells and to calculate percentage inhibition [42].

(2) $$\begin{array}{c} \text{Inhibitory activity}\left(\%\right)=\frac{{\text{CPM}}_{\left(\text{Control group}\right)}-{\text{CPM}}_{\left(\text{Test group}\right)}}{{\text{CPM}}_{\left(\text{Control group}\right)}}\times 100. \end{array}$$

#### 3.1.6. Cytotoxicity Essay

A cell suspension (100 *μ*L) of concentration 6 × 10^4^ cell/mL was introduced into flat-bottomed plates and incubated in 5% CO_2_ at 37°C for 24 hours. The test wells received the extract (3.125, 6.25, 12.5, 25, and 50 *μ*g/mL) and the complete DMEM for a final volume of 200 *μ*L. The positive control wells received only the complete DMEM, whereas the negative control wells received Triton X-100 (0.5%). The plate was incubated (CO_2_, 37°C, 48 h), then the supernatant was removed, 50 *μ*L of MTT (0.5 mg/mL) diluted in PBS of 5 mg/mL was then introduced into each well, and the plate was further incubated for 4 h. One hundred microliters (100 *μ*L) of DMSO was added to each well after aspiration of MTT, and the plate was stirred (for 10 to 15 min) with an orbital shaker. The absorbance was read at 540 nm using a spectrophotometer, and the percent inhibition was determined [44].

(3) $$\begin{array}{c} \%\text{Inhibition}=100-\frac{{\text{OD}}_{\text{test group}}-{\text{OD}}_{\text{blank}}}{{\text{OD}}_{\text{Control group}}-OD_{\text{blank}}}\times 100. \end{array}$$

## 4. *In Vivo* Assay

### 4.1. Animals

For this test, Wistar rats of both sexes with an average mass of 225 g and an age between 2.5 and 3 months were used. The Animal House of the Department of Animal Biology of the Faculty of Science of the University of Dschang in Cameroon served as a framework for animal husbandry. The animals were fed a standard diet for rodents and had water *ad libitum*. The animals were then divided into groups according to their weight and left for acclimation for 48 hours before the start of any experiment.

The experimental procedures were approved by the local ethics committee and complied with the guidelines for the study of pain in awake animals, published by the NIH Publication No. 85-23 “Principles of Animal Protection,” “Laboratory,” Study of Pain, Ministry of Scientific Research and Technology, which adopted the European Union guidelines on animal care and experimentation (Conseil CEE 86/609).

### 4.2. Colitis Induction

Forty-two (42) rats were divided according to their weight into 7 groups of 6 rats each. With the exception of group I, considered as a neutral control, which did not receive any DSS solution, all the other groups received the double DSS solution. From the 6th day after the commencement of colitis induction, all animals were treated as follows: group I (neutral control) received distilled water (1 ml/100 g bw), group II (negative control) received a solution of DMSO (5%) + PBS, group III (positive control) received a solution of prednisolone (4 mg/kg), groups IV and V (treated) were given the aqueous extract (125 and 250 mg/kg), and groups VI and VII (treated) received the methanol extract (125 and 250 mg/kg). All treatments were given orally from the 6th day to the 15th day after the start of induction.

### 4.3. Evaluation of Body Weight and Stool Condition

Body weight (estimated as relative body weight) and stool status (estimated as a Bristol stool score [45]) of all animals were evaluated daily, from day 0 before the start of induction of colitis to the 15^th^ day following start of induction of colitis.

### 4.4. Hematology, Biochemical Parameters, Weight, and Organ Length

On the nineteenth day, all the animals received thiopental (50 mg/kg, 0.1 ml/100 g bw) intraperitoneally for anesthesia, and then the blood was taken by catheterization of the abdominal artery and introduced into tubes with anticoagulant (heparin) for the estimation of red blood cells, white blood cells, and blood platelets. Then the liver and spleen were isolated, freed from fat, and then weighed using a scale. The complete colon located between the ileocolic junction and the anus was also rapidly removed, opened longitudinally along the mesenteric fixation line, washed with saline in an ice-water tank, and lightly dried with filter paper; then, the length and weight of all two points were measured. Subsequently, the colon was cut into two parts: one part was stored in 10% PBS-buffered formalin for histological analysis while the other was ground in phosphate buffer for evaluation of oxidative stress parameters including catalase (CAT), glutathione (GSH), superoxide dismutase (SOD), malondialdehyde (MDA), and nitrogen monoxide (NO).

### 4.5. Macroscopic and Microscopic Evaluation of the Colon

Immediately after isolating (between the ileocolic junction and the anus) and washing the complete colon, photographs were taken using a Canon camera (Cyber-shot, 7.2 megapixels, China). Colon samples fixed in 10% PBS-buffered formalin were fixed, cut into 5 *μ*m pieces each, and stained with hematoxylin-eosin (H&E) for microscopic evaluation.

### 4.6. Statistical Analysis

The values are expressed as mean ± SEM. Statistical analysis between the means was performed using one-way ANOVA, followed by the Tukey posttest (hematology, length, and weight of the organs, biochemical parameters), two-way ANOVA, and the Bonferroni posttest (body weight and stools condition). Significance was statistically acceptable at a level of *P* < 0.05. The Graph Pad InStat software was used for all data analysis.

## 5. Results

### 5.1. Chemical Composition

The aqueous and methanol extracts of *A. boonei* were subjected to phytochemical screening. Our results indicated that both extracts contain 6 classes of secondary metabolites including tannins, saponins, alkaloids, steroids, flavonoids, and triterpenoids (Table 1).

## 6. *In Vitro* Test

### 6.1. *A. boonei* Extracts on Intracellular ROS Production

The aqueous and methanol extracts of *A. boonei* were evaluated for their ability to inhibit the production of intracellular ROS in whole blood, by PMN and by macrophages after stimulation of cells with zymosan A. The results showed that the aqueous extract inhibited the production of ROS with IC_50_ values of 10.04 ± 0.55 *μ*g/mL (whole blood), 8.80 ± 0.24 *μ*g/mL (PMNs), and 9.48 ± 1.24 *μ*g/mL (macrophage). Similarly, the methanol extract also inhibited the production of intracellular ROS in whole blood, by PMN and by macrophages with IC_50_ values of 7.08 ± 0.24 *μ*g/mL, 5.47 ± 0.03 *μ*g/mL, and 5.98 ± 0.03 *μ*g/mL, respectively. Ibuprofen used as a reference substance inhibited intracellular ROS production with IC_50_ values of 15.81 ± 0.22 *μ*g/mL (whole blood), 15.20 ± 0.64 *μ*g/mL (PMNs), and 15.69 ± 1.45 *μ*g/mL (macrophages) (Table 2).

### 6.2. *A. boonei* Extracts on Extracellular ROS Production

PMA has been used to stimulate the production of extracellular ROS in whole blood, PMNs, and macrophages in the presence of various extracts of *A. boonei*. The results showed that extracellular ROS production in whole blood, by PMN and by macrophages was inhibited by both extracts of *A. boonei* with IC_50_ values of 11.58 ± 0.65 *μ*g/mL, 8.26 ± 0.22 *μ*g/mL, and 7.16 ± 0.13 *μ*g/mL for the aqueous extract and 7.12 ± 0.19 *μ*g/mL, 6.60 ± 0.16 *μ*g/mL, and 7.08 ± 0.27 *μ*g/mL for the methanol extract. Ibuprofen inhibited extracellular ROS production with a maximum IC_50_ value of 15.55 ± 0.54 *μ*g/mL (for PMNs) (Table 2).

### 6.3. *A. boonei* Extracts on Cytokines and Prostaglandin E2

**Figure 1:**
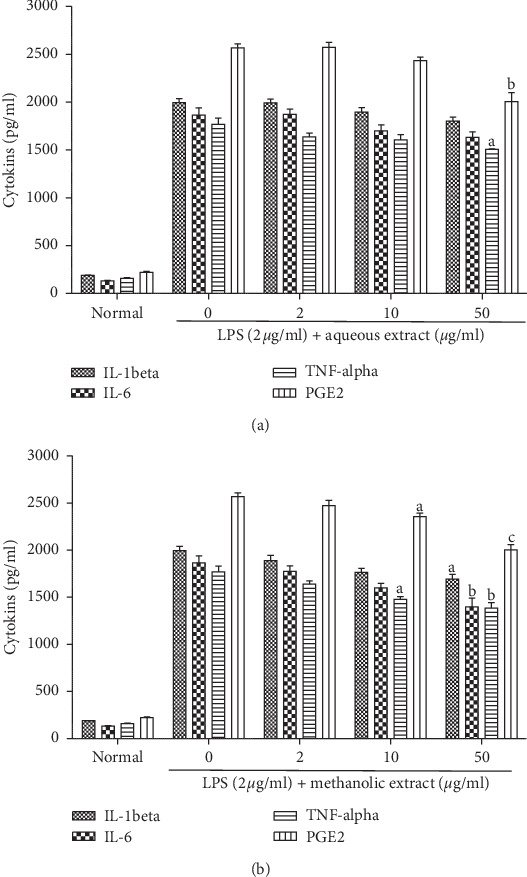
Effect of extracts of *Alstonia boonei* on cytokines and prostaglandin E2 secretion levels by mice macrophages activated by LPS. Data represent mean ± SD of triplicates and are analyzed by one-way ANOVA followed by Tukey post hoc test; ^a^*P* < 0.05, ^*b*^*P* < 0.01, ^*c*^*P* < 0.001 when compared with nontreated group (0 mg/kg).

The effects of the aqueous and methanol extracts of *A. boonei* on the production of TNF-*α*, IL-1*β*, IL-6, and PGE2 by LPS-activated macrophages were evaluated. It appeared that the concentrations of 2 *μ*g/mL and 10 *μ*g/mL of the aqueous extract and 2 *μ*g/mL of the methanol extract did not cause any significant effect on the production of TNF-*α*, IL-1*β*, IL-6, and PGE2. At the concentration of 50 *μ*g/ml, the aqueous extract significantly inhibited the production of TNF-*α* (*P* < 0.05) and of PGE2 (*P* < 0.01), whereas the methanol extract at the same concentration significantly inhibited the production of TNF-*α* (*P* < 0.01), IL-1*β* (*P* < 0.05), IL-6 (*P* < 0.01), and PGE2 (*P* < 0.001) (Figure 1).

### 6.4. *A. boonei* Extracts on the T-Cell Proliferation Assay

The aqueous and methanol extracts of *A. boonei* were tested on the proliferation of T-cells. It is clear from this test that the aqueous extract, methanol extract, and prednisolone inhibited the cell proliferation with IC_50_ values of 46.00 ± 1.17 *μ*g/mL, 8.19 ± 1.10 *μ*g/mL, and <3.10 *μ*g/mL, respectively (Table 2).

### 6.5. Cytotoxicity of *A. boonei* Extracts on 3T3 Cells

Aqueous and methanol extracts of *A. boonei* showed potential inhibitory effects in various immunoassays when they were tested for possible toxic effects. It was noticed that compared to cyclohexamide (which is considered to be a toxic substance with an IC_50_ value of 0.10 ± 0.13 *μ*g/mL), the aqueous extract was found to have no toxic effect with an IC_50_ value of 87.00 ± 1.23 *μ*g/mL, while the methanol extract had a very moderate level of toxicity with an IC_50_ value of 37.03 ± 0.56 *μ*g/mL (Table 2).

## 7. *In vivo* Assay

### 7.1. *A. boonei* Extracts on Body Weight

**Figure 2:**
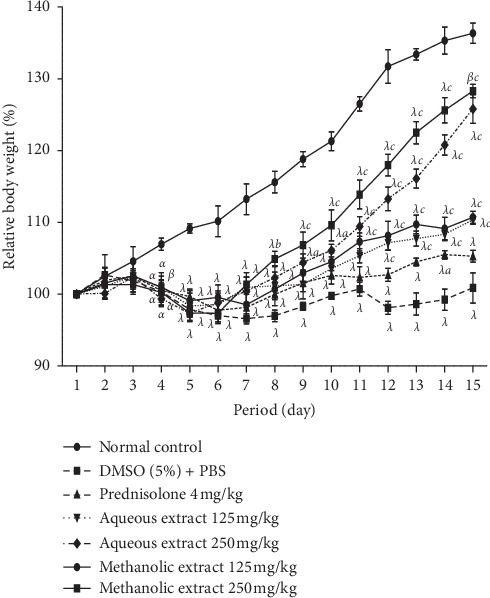
Effects of aqueous and methanol stem-bark extracts of *A. boonei* on relative body weight in DSS-induced colitis in rats. Values are expressed as mean ± SEM for six animals and analyzed by two-way ANOVA followed by Bonferroni post hoc test; ^a^*P* < 0.05, ^*b*^*P* < 0.01, ^*c*^*P* < 0.001: significant difference compared with DMSO (5 %) + PBS; ^*α*^*P* < 0.05, ^*β*^*P* < 0.01, ^*λ*^*P* < 0.001: significant difference compared to normal control.

Figure 2 shows that the body weight of all animals treated with DSS decreased significantly (*P* < 0.05, *P* < 0.001) at days 3 and 4 compared to animals in the neutral control group. At a dose of 125 mg/kg, the aqueous and methanol extracts of *A. boonei* significantly increased (*P* < 0.05, *P* < 0.01) the body weight of the treated animals on the 7^th^ day. In addition, at the dose of 250 mg/kg, the increase in body weight was significant on day 3 (*P* < 0.05) for the aqueous extract and on day 6 (*P* < 0.01) for the methanol extract.

### 7.2. *A. boonei* Extracts on Stool Condition

**Figure 3:**
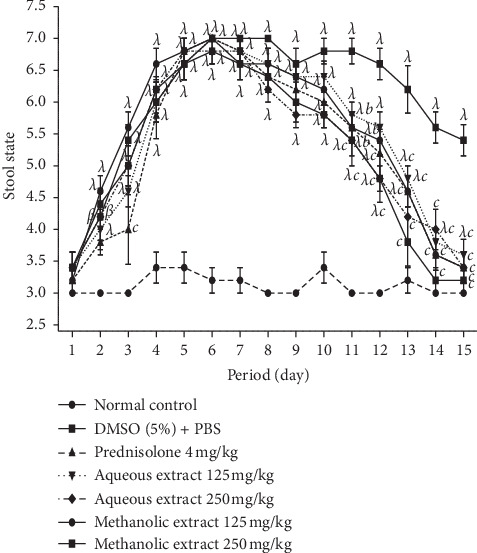
Effects of aqueous and methanol stem-bark extracts of *A. boonei* on stool state in DSS-induced colitis in rats. Values are expressed as mean ± SEM for six animals and analyzed by two-way ANOVA followed by Bonferroni post hoc test; ^a^*P* < 0.05, ^*b*^*P* < 0.01, ^*c*^*P* < 0.001: significant difference compared with DMSO (5 %) + PBS; ^*α*^*P* < 0.05, ^*β*^*P* < 0.01, ^*λ*^*P* < 0.001: significant difference compared to normal control.

Figure 3 shows the stool condition of the animals treated with *A. boonei* aqueous (A) and methanol (B) extracts (125 and 250 mg/kg) and prednisolone (4 mg/kg) according to the stool diagram of Bristol. It appeared that the score increased significantly (*P* < 0.05, *P* < 0.01, *P* < 0.001) in all animals treated on day 2 after induction of colitis compared to animals in the neutral control group. On the 10^th^ day, 5 days after the start of treatment, the score of the animals treated with the different extracts and with prednisolone was significantly (*P* < 0.05, *P* < 0.01, *P* < 0.001) improved compared to that of the animals of negative control group (DMSO 5% + PBS). In addition, the score of all treated animals was almost similar to that of the neutral control group between day 14 and day 15.

### 7.3. *A. boonei* Extracts on Some Hematological Parameters

Table 3 shows the hematological variations of the animals after 15 days of treatment. It appeared that the level of red blood cells decreased significantly, while the levels of white blood cells and blood platelets increased significantly in the animals having received the mixture DMSO-PBS compared to the animals of the neutral control group. Treatment with the aqueous and methanol extracts of *A. boonei*, as well as prednisolone, resulted in a significant increase in red blood cell count and a significant decrease in white blood cells and blood platelets compared to animals in the negative control group.

### 7.4. *A. boonei* Extracts on Mass of Some Selected Organs

Table 4 shows that the treatment with DSS resulted in a nonsignificant reduction in the masses of the colon, the liver, and the spleen compared to those of the animals of the neutral control group. Furthermore, the administration of the aqueous and methanol extracts of *A. boonei* led to a nonsignificant increase in the masses of the organs studied compared to those of the animals in the negative control group.

### 7.5. *A. boonei* Extracts on Colon Length

**Figure 4:**
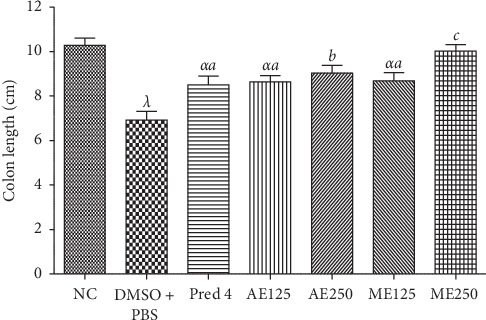
Effects of the aqueous and methanol stem-bark extracts of *A. boonei* on colon length in DSS-induced colitis in rats. Values are expressed as mean ± SEM for six animals and analyzed by one-way ANOVA followed by Tukey post hoc test; ^a^*P* < 0.05, ^*b*^*P* < 0.01, ^*c*^*P* < 0.001: significant difference compared with DMSO + PBS; ^*α*^*P* < 0.05: significant difference compared to normal control.

Figure 4 shows the variation in colon length in animals after 15 days of observation. It appeared that the DSS caused a significant decrease in the length of the colon in the animals treated with the DMSO-PBS mixture compared to that of the animals of the neutral control group. Both extracts and prednisolone caused a significant increase in colon length in all treated animals compared to animals in the negative control group. This increase became identical to that of animals in the neutral control group at a dose of 250 mg/kg.

### 7.6. *A. boonei* Extracts on Few Biochemical Parameters

**Figure 5:**
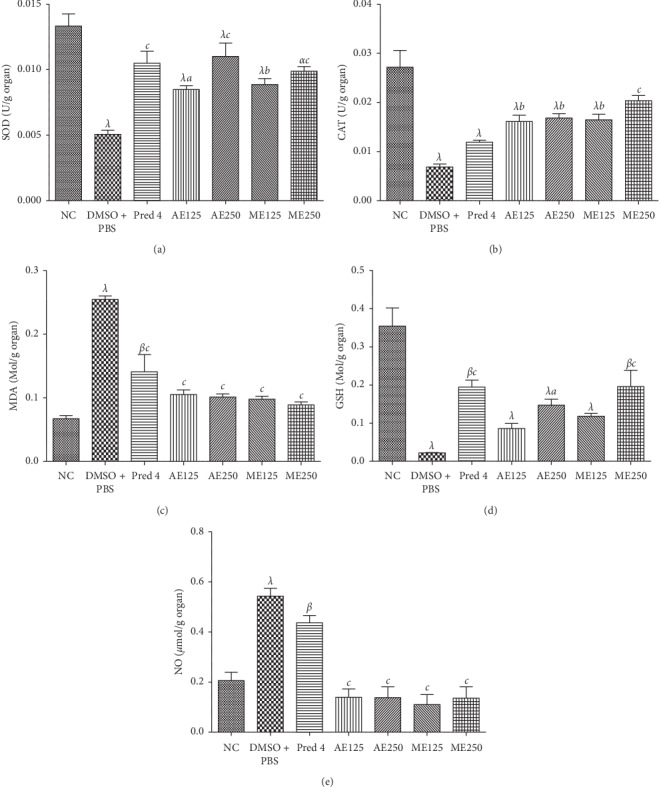
Effects of the aqueous (AE) and methanol (ME) extracts of *A. boonei* on some parameters of oxidative stress in DSS-induced colitis in rats. Values are expressed as mean ± SEM for six animals and analyzed by one-way ANOVA followed by Tukey post hoc test; ^a^*P* < 0.05, ^*b*^*P* < 0.01, ^*c*^*P* < 0.001: significant difference compared with distilled DMSO + PBS; ^*α*^*P* < 0.05, ^*β*^*P* < 0.01, ^*λ*^*P* < 0.001: significant difference compared to normal control.

Figure 5 shows the effects of the aqueous and methanol extracts of *A. boonei* on SOD, CAT, MDA, GSH, and NO levels. It was found that, after DSS administration, SOD and CAT activity decreased significantly (*P* < 0.05, *P* < 0.01, *P* < 0.001) in all animals. Administration of the different extracts at doses of 125 and 250 mg/kg significantly (*P* < 0.01, *P* < 0.001) increased SOD and CAT activity in all treated animals compared to animals that received DMSO (5%) + PBS. Prednisolone (4 mg/kg) provoked a significant (*P* < 0.001) increase in SOD and a not significant one in CAT.

In addition, the administration of DSS resulted in a significant increase (*P* < 0.001) in MDA and NO levels, followed by a significant decrease (*P* < 0.001) in GSH levels in DMSO (5%) + PBS-treated animals compared to the neutral control groups. The aqueous and methanol extracts (125 and 250 mg/kg) as well as prednisolone (4 mg/kg) resulted in a significant decrease (*P* < 0.001) in MDA and NO levels compared to the animals in the negative control groups. In addition, only the 250 mg/kg dose of the aqueous and methanol extracts resulted in a significant (*P* < 0.05, *P* < 0.001) increase in GSH compared to the control negative group.

### 7.7. Histopathological Evaluation

**Figure 6:**
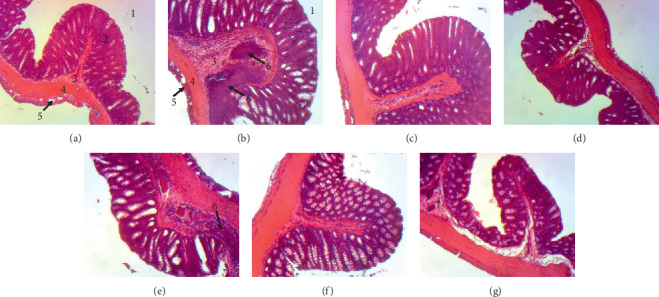
Effects of the aqueous and methanol stem-bark extracts of *A. boonei* on microphotographs of colon (hematoxylin-eosin, x100). (a) Normal control; (b) negative control; (c) prednisolone (4 mg/kg); (d) aqueous extract (125 mg/kg); (e) aqueous extract (250 mg/kg); (f) methanol extract (125 mg/kg); (g) methanol extract (250 mg/kg). 1: intestinal lumen, 2: mucosa, 3: submucosa, 4: muscularis, 5: serosa, 6: lymphocytic hyperplasia.

Histological analysis of colon tissues revealed untreated (negative control) alterations such as loss of tissue architecture, mucosal infiltrate, thickening of the submucosal muscle, and hemorrhage in the mucosa crypts after DSS injection in animals treated with all doses (Figures 6(d)–6(g)) of extracts of *A. boonei* compared to the control group (Figure 6(b)). This effect was also observed for prednisolone used as a reference product (Figure 6(c)).

### 7.8. Macroscopic Assessment of Colon Damage

**Figure 7:**
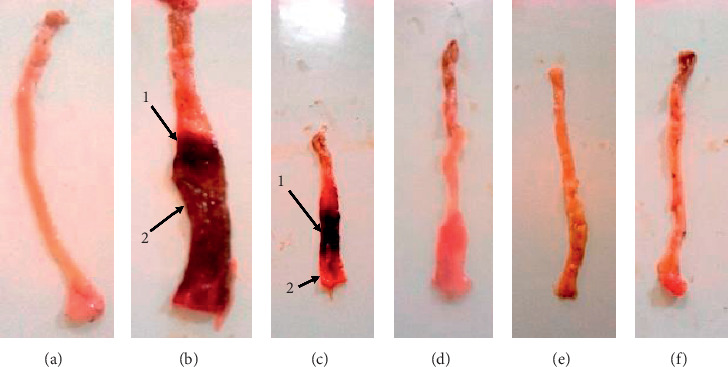
Photographs of colon following treatment with the aqueous and methanol stem-bark extracts of *A. boonei.* (a) Normal control; (b) negative control; (c) DMSO (5 %); (d) prednisolone (4 mg/kg); (e) aqueous extract (250 mg/kg); (f) methanol extract (250 mg/kg). 1: bleeding, 2: edema.

Figure 7 shows the macroscopic presentation of the intestine of animals after induction of DSS-induced colitis in the rats. It was found that DSS causes bleeding development and edema in animals treated with DMSO (5%) + PBS (Figures 7(b) and 7(c)). On the other hand, the animals that received the aqueous and methanol extracts of *A. boonei* as well as prednisolone showed no bleeding or edema (Figures 7(d)–7(f)).

## 8. Discussion

Ulcerative colitis, which is one of the major forms of inflammatory bowel disease, is a chronic inflammatory disease characterized by inflammation, mucosal ulcerations, tissue cell influx, and exaggerated production of many proinflammatory mediators [46, 47]. Several animal models have been developed to allow the understanding of inflammatory bowel diseases and to promote the development of new management strategies for patients suffering from these diseases [48]. One of the most widely used models is the administration of DSS in drinking water which causes chronic intestinal inflammation in animals, thus allowing important observations on the immune system and the study of many mediators and the important cellular influx involved in the chronic development of inflammatory diseases of the intestine [49, 50]. The repeated administration of DSS causes an amplification of the inflammatory response in ulcerative colitis with an exaggerated production of ROS, TNF-alpha, IL-1 beta, and IL-6 which will be responsible for the severity of the colitis and the appearance of symptoms related to inflammatory bowel diseases [51, 52]. Similarly, during the development of colitis, elevated levels of proinflammatory cytokines are accompanied by elevated levels of prostaglandin and eicosanoids as a result of increased expression of COX-2 [53].

In this study, *in vitro* tests demonstrated that the aqueous and methanol extracts of *A. boonei* significantly inhibited the release of TNF-*α*, IL-6, IL-1*β*, and PGE2. The results also suggest that the inhibitory activity of aqueous and methanol extracts of *A. boonei* on the production of these proinflammatory cytokines has clinical significance. This inhibitory activity of the release of TNF-*α*, IL-6, IL-1*β*, and PGE2 by *A. boonei* extracts indicates that this plant is rich in compounds having an anti-inflammatory potential with a possible capacity to intervene in the immune response, since the inhibitory effects of certain compounds on the production of TNF-*α*, IL-6, IL-1*β*, and/or PGE2 is an important marker taking into account their major role in growth, differentiation, and death of immune cells, just as in the effective treatment of most inflammatory diseases [54].

In many inflammatory states, there is significant polymorphonuclear cell infiltration followed by an increase in the chemokine (CXCL1/KC) required for migration of neutrophils to the lesional focus [55, 56]. Thus, the effects of the aqueous and methanol extracts of *A. boonei* have been evaluated on another important aspect of the immune response which is cell proliferation. It is apparent from the results that the extracts significantly inhibited cell proliferation in a dose-response manner. It is important to suggest that the inhibitory activity of extracts on this cell proliferation could be the consequence of a significant reduction in the production of proinflammatory cytokines. The results also suggest that the compounds present in *A. boonei* extracts are capable of significantly influencing the immune response of phagocytes and monocytes at different stages, since macrophages and neutrophils play a very important role in mucosal inflammation [57, 58], given that macrophages are an important source of proinflammatory cytokines that regulate epithelial barrier and/or cell proliferation while neutrophils contribute significantly to tissue damage [59]. This antiproliferative activity of the aqueous and methanol extracts of *A. boonei* could be associated with the presence of compounds such as alpha-amyrin and beta-amyrin which are compounds possessing antiproliferative properties [60] and are present in the extracts of *A. boonei*.

Clinically, it is known that people suffering from ulcerative colitis have overproduction of ROS that would be responsible for lipid peroxidation, inhibition of the antioxidant capacity of cells, and attack of tissue proteins and also of DNA [10–12]. The aqueous and methanol extracts of *A. boonei* showed good antioxidant capacity in this study, significantly inhibiting the production of extra- and intracellular ROS in whole blood and various phagocytic cells (neutrophils and macrophages). As we know that, during the inflammatory reaction, the NF-kB/AP-1 axes play an important role in the production of TNF-*α*, NO, IL-1*β*, and PGE2 [61, 62] and that the phosphorylation of NF-kB factor by many compounds significantly decreases the T-cell proliferation process [63], it is possible that the inhibitory properties of the aqueous and methanol extracts of *A. boonei* are associated with an action on the factor NF-kB. Since this study showed that this plant was rich in flavonoids, saponins, steroids, and terpenoids and several studies have shown that several terpenoids and flavonoids have inhibitory effects on NF-kB factor phosphorylation [64, 65], this justified their anti-inflammatory and antiproliferative properties. In addition, the work of Vitor et al. [66] has shown that the alpha- and beta-amyrin compounds that have been found in *A. boonei* extracts are able to inhibit the phosphorylation of NF-kB factor just as the expression of COX-2. Moreover, Beserra et al. [67] reported that lupeol which has also been isolated from *A. boonei* extract has an inhibitory effect on NF-kB factor phosphorylation.

In the present study, the cytotoxic activity of various extracts of *A. boonei* was evaluated on a 3T3 cell line. The aqueous extract showed no toxic effect with an IC_50_ value equal to 87.80 *μ*g/ml, while the methanol extract with an IC_50_ value equal to 37.03 *μ*g/ml showed a very low toxicity. Nevertheless, in order to better estimate the toxicity of a compound in humans and to identify toxic products, *in vitro* cytotoxicity tests are generally used [42], depending on the substance tested [68]. In an *in vitro* test, which can increase the reliability of the results obtained, more than one test should be required to determine cell viability. However, Nkono Ya Nkono et al. [38] and Enechi et al. [37] showed that, during acute and/or subchronic treatment, the methanol and aqueous extracts of *A. boonei* did not cause death in rats during toxicity with an LD50 > 5 g/kg body weight, which classifies this plant as a nontoxic plant [69].

One of the animal models of experimentally induced colitis is the addition of DSS to drinking water which can be induced in hamsters, rats, and mice [49]. During the development of this model, the pathological changes are similar to those observed in inflammatory bowel diseases in humans [17]. The most common symptoms are bloody diarrhea, weight loss, shortening of the colon, ulceration of the mucosa, and neutrophil infiltration. Several authors have shown that, during the course of DSS-induced colitis, the onset of various symptoms is accompanied by an increase in oxidative stress, an increase in inflammatory markers (TNF-alpha, IL-1, NO, and NF-kB), and an increase in the expression of iNOS [70–73]. In the present study, the aqueous and methanol extracts of *A. boonei* showed significant activity against all clinical symptoms of colitis. They significantly increased body weight in all treated animals, improved stool condition, and increased colon length. This important activity of *A. boonei* extracts is probably due to the presence of compounds such as lupeol, alpha-amyrin, and beta-amyrin in this plant, since these compounds have shown their ability to remarkably inhibit colitis induced in mice by TNBS by reducing inflammatory cytokines and COX-2 expression and/or inhibition of NF-kB factor phosphorylation [66, 67].

In pathogenicity of inflammatory bowel disease, the overproduction of free radicals, ROS, and even proinflammatory cytokines can be explained by the multiple interactions that exist between environmental, genetic, and immune factors. Thus, we can observe the installation of a cascade of inflammatory processes accompanied by an oxidative stress resulting from an imbalance between the prooxidant and antioxidant systems in favor of the prooxidant one [74, 75]. Decreased antioxidant capacity contributes to the pathogenesis of colitis and other inflammatory disorders [76]. The first endogenous defense line, capable of preventing oxidative damage, includes enzymatic (SOD and CAT) and nonenzymatic antioxidants (GSH, MDA, and NO) [77]. Malondialdehyde (MDA) is a major end product of lipid peroxidation. Increasing MDA content may contribute to increased free radical generation and reduced activity of antioxidant defense systems [17]. In this study, DSS resulted in a marked increase in lipid peroxidation, expressed as MDA content. At the same time, SOD is a metalloprotein that catalyzes the dismutation of two superoxide radicals to form hydrogen peroxide and molecular oxygen [17]. The decrease in SOD activity and catalase may be due to the increased generation of reactive oxygen species, such as superoxide and hydrogen peroxide. Glutathione (GSH) is an important intracellular antioxidant that plays an important role in protecting cells against oxidative stress [17]. Under physiological conditions, NO, which plays an important role in the regulation of the motility and cytoprotection processes of the large intestine, is synthesized from L-arginine by the constituent forms of NO synthase [78]. In addition, some authors have found that animals with DSS-induced colitis have significantly increased level of NO in the intestine [79]. These results show that a treatment with the aqueous and methanol extracts of *A. Boonei* (125 and 250 mg/kg) significantly reduced levels of MDA, GSH, and NO; this decrease could be attributed to the free radical scavenging potential of the compounds present in these extracts. At the same time, treatment with both extracts significantly attenuated SOD and CAT levels. Both extracts were rich in polyphenols which are compounds that exert some of their antioxidant effects by promoting the secretion of antioxidant enzymes (e.g., SOD and CAT) and by inducing HO-1-related detoxification enzymes. Glutathione is involved in the detoxification of xenobiotics [80]. In addition, aqueous and methanol extracts contain flavonoids and saponins that exert their antioxidant power by free radical scavenging [81, 82]. These results are also in agreement with those reported by Akinmoladun et al. [30] on the in vitro antioxidant properties of aqueous and methanolic extracts of *A. boonei*.

Relapses observed in stool consistency of all groups are common in ulcerative colitis. However, normal stool consistency was observed and maintained in the treated groups on day 13. This may be due to the effect of administration of *A. boonei* extracts, as this has been reported earlier by us to possess antidiarrheal properties [83]. Both extracts of *A. boonei* significantly increased the length of the colon in the treated groups, effects that were similar to those of prednisolone. This could be attributed to the antiulcer properties of *A. boonei* extracts by the action on mucosal defense factors. This is justified by a previous work reported by Okoye et al. [35] on this plant showing antiulcer properties on the ulcer models induced by pyloric ligation and acetic acid in rats, an activity that is related to its antiproliferative and antioxidant properties. Administration of DSS to mice or rats in their drinking water for a short period of time resulted in limited inflammation in the colon characterized by erosions/ulcers, loss of crypts, and granulocyte infiltration [49, 51]. Micrographs of histological sections showed that inflammation, ulcer, and edema decreased in animals treated with the aqueous and methanol extracts of *A. boonei* and prednisolone. Complete recovery was observed earlier in the groups treated with the methanol and aqueous extracts, similarly to the normal control group, and it was evident by intact mucosa. These results show that *A. boonei* is effective in reducing macroscopic and histological damage in the same way as the reference drug, prednisolone, and that it could be useful in the treatment of colitis.

## 9. Conclusion

Based on the results, it could be concluded that the administration of the aqueous and methanol extracts of *A. boonei* significantly reduces the symptoms and morphological and biochemical characteristics of ulcerative colitis. Decreasing the release of inflammatory markers with modulation of oxidative/antioxidant balance in colon tissues could probably explain the protective role of *A. boonei* extracts. However, further studies are needed to evaluate whether similar efficacy can be achieved in other models of experimental colitis simulating human inflammatory bowel disease.

## Figures and Tables

**Table 1:** Phytochemical profile of *A. boonei*.

Extracts	Phytochemical compounds					
	1	2	3	4	5	6
Aqueous extract	+	+	+	_+_	+	+
Methanolic extract	+	+	+	+	+	+

−: absent; +: present; 1: tannins; 2: saponins; 3: alkaloids; 4: steroids; 5: flavonoids; 6: triterpenoids.

**Table 2:** IC_50_ values of aqueous and methanolic stem-bark extracts of *A. boonei* on human whole blood evaluated by luminol and/or lucigenin amplified chemoluminescence, on T-cells proliferation, and on cytotoxicity on 3T3 cells.

Treatment	Oxidative burst (IC_50_ *μ*g/ml)						T-cells proliferation (IC_50_ *μ*g/ml)	Cytotoxicity on 3T3 cells (IC_50_ *μ*g/ml)
	Luminol/zymosan			PAM/lucigenin				
	WB	PMNs	MQ	WB	PMNs	MQ		
Aqueous extract	10.04 ± 0.55	8.80 ± 0.24	9.48 ± 1.24	11.58 ± 0.65	8.26 ± 0.22	7.16 ± 0.13	26.46 ± 1.17	87.80 ± 1.23
Methanolic extract	7.08 ± 0,24	5.47 ± 0.03	5.98 ± 0.03	7.12 ± 0.19	6.60 ± 0.16	7.08 ± 0.27	8.19 ± 1.10	37.03 ± 0.56
Ibuprofen	15.81 ± 0.22	15.20 ± 0.64	15.69 ± 1.45	17.83 ± 0.16	15.55 ± 0.54	16.57 ± 0.54	—	—
Prednisolone	—	—	—	—	—	—	<3.10	—
Cyclohexamide	—	—	—	—	—	—	—	0.10 ± 0.13

The IC_50_ values are presented as mean ± SD of triplicates. WB: whole blood, PMNs: polymorphonuclear neutrophils, MQ: mice peritoneal macrophages.

**Table 3:** Effects of the aqueous and methanol stem-bark extracts of *A. boonei* on hematology in DSS-induced colitis in rats.

Treatment	Dose (mg/kg)	RBCs (million/*μ*l)	WBCs (10^9^/L)	Platelets (10^9^/L)
Normal control	—	8.16 ± 0.15	4.90 ± 0.42	327.20 ± 13.53
DMSO (5 %) + PBS	—	6.08 ± 0.26^*λ*^	10.45 ± 0.37^*λ*^	744.20 ± 61.36^*λ*^
Prednisolone	4	7.95 ± 0.40^c^	8.24 ± 1.17^*α*^	520.80 ± 57.62^*α*a^
Aqueous extract	125	7.10 ± 0.10^*α*a^	7.18 ± 0.79^a^	460.20 ± 42.13^c^
	250	7.64 ± 0.16^c^	6.38 ± 0.70^b^	468,60 ± 36.56^b^
Methanol extract	125	7.37 ± 0.09^b^	6.46 ± 0.38^b^	490.20 ± 28.85^b^
	250	7.67 ± 0.12^c^	6.22 ± 0.43^b^	410.60 ± 35.87^c^

Values are expressed as mean ± SEM for six animals and analyzed by one-way ANOVA followed by Tukey post hoc test; ^a^*P* < 0.05, ^*b*^*P* < 0.01, ^*c*^*P* < 0.001: significant difference compared with DMSO + PBS; ^*α*^*P* < 0.05, ^*λ*^*P* < 0.01: significant difference compared to normal control.

**Table 4:** Effects of the aqueous and methanol stem-bark extracts of *A. boonei* on colon, liver, and spleen mass in DSS-induced colitis in rats.

Treatment	Dose (mg/kg)	Mass (g)		
		Colon	Liver	Spleen
Normal control	—	176 ± 0,09	5.43 ± 0.10	0,66 ± 0,06
DMSO (5 %) + PBS	—	1,42 ± 0,05	4,87 ± 0,09	0,55 ± 0,02
Prednisolone	4	1,67 ± 0,05	5,08 ± 0,49	0,51 ± 0,01
Aqueous extract	125	1,65 ± 0,07	5,60 ± 0,52	0,64 ± 0,05
	250	1,43 ± 0,12	5,01 ± 0,32	0,64 ± 0,02
Methanol extract	125	1,45 ± 0,08	5,32 ± 0,21	0,64 ± 0,04
	250	1,60 ± 0,07	5,61 ± 0,24	0,66 ± 0,03

Values are expressed as mean ± SEM for six animals and analyzed by one-way ANOVA followed by Tukey post hoc test.

## Data Availability

All data supporting our findings are adequately contained within the manuscript.

## References

[1] Garrett W. S., Gordon J. I., Glimcher L. H. (2010). Homeostasis and inflammation in the intestine. *Cell*.

[2] Matricon J., Barnich N., Ardid D. (2010). Immunopathogenesis of inflammatory bowel disease. *Self/Nonself*.

[3] Morampudi V., Bhinder G., Wu X. (2014). DNBS/TNBS colitis models: providing insights in to inflammatory bowel disease and effects of dietary fat. *Journal of Visualized Experiments*.

[4] Zhao H.-Y., Yang G.-T., Sun N.-N., Kong Y., Liu Y.-F. (2017). Efficacy and safety of stellate ganglion block in chronic ulcerative colitis. *World Journal of Gastroenterology*.

[5] Chan Y., Lim E. T., Sandholm N. (2014). An excess of risk-increasing low-frequency variants can be a signal of polygenic inheritance in complex diseases. *The American Journal of Human Genetics*.

[6] Kim D. H., Cheon J. H. (2017). Pathogenesis of inflammatory bowel disease and recent advances in biologic therapies. *Immune Network*.

[7] Park J. H., Peyrin-Biroulet L., Eisenhut M., Shin J. I. (2017). IBD immunopathogenesis: a comprehensive review of inflammatory molecules. *Autoimmunity Reviews*.

[8] Carty E., De B. M., Feakins R. M., Rampton D. S. (2000). Measurement of in vivo rectal mucosal cytokine and eicosanoid production in ulcerative colitis using filter paper. *Gut*.

[9] Lih-Brody L., Powell S. R., Collier K. P. (1996). Increased oxidative stress and decreased antioxidant defenses in mucosa of inflammatory bowel disease. *Digestive Diseases and Sciences*.

[10] Tahan G., Aytac E., Aytekin H. (2011). Vitamin E has a dual effect of anti-inflammatory and antioxidant activities in acetic acid-induced ulcerative colitis in rats. *Canadian Journal of Surgery*.

[11] Pravda J. (2005). Radical induction theory of ulcerative colitis. *World Journal of Gastroenterology*.

[12] Tüzün A., Erdil A., İnal V. (2002). Oxidative stress and antioxidant capacity in patients with inflammatory bowel disease. *Clinical Biochemistry*.

[13] Sartor R. B. (1997). Pathogenesis and immune mechanisms of chronic inflammatory bowel diseases. *American Journal of Gastroenterology*.

[14] Andoh A., Yagi Y., Shioya M., Nishida A., Tsujikawa T., Fujiyama Y. (2008). Mucosal cytokine network in inflammatory bowel disease. *World Journal of Gastroenterology*.

[15] Bertevello P. L., Logullo A. F., Nonogaki S. (2005). Immunohistochemical assessment of mucosal cytokine profile in acetic acid experimental colitis. *Clinics (Sao Paulo)*.

[16] Mehta S. J., Silver A. R., Lindsay J. O. (2013). Review article: strategies for the management of chronic unremitting ulcerative colitis. *Aliment Pharmacology Therapy*.

[17] Somani S. J., Badgujar L. B., Sultariya B. K., Saraf M. N. (2014). Protective effect of Dillenia indica L. on acetic acid induced colitis in mice. *Indian Journal of Experimental Biology*.

[18] Vickers A. D., Ainsworth C., Mody R. (2016). Systematic review with network meta-analysis: comparative efficacy of biologics in the treatment of moderately to severely active ulcerative colitis. *PLoS One*.

[19] Testa A., Castiglione F., Nardone O. M., Colombo G. L. (2017). Adherence in ulcerative colitis: an overview. *Patient Preference and Adherence*.

[20] Fiorino G., Bonovas S., Cicerone C. (2017). The safety of biological pharmacotherapy for the treatment of ulcerative colitis. *Expert Opinion on Drug Safety*.

[21] Farghaly H. S., Thabit R. H. (2014). L-arginine and aminoguanidine reduce colonic damage of acetic acid-induced colitis in rats: potential modulation of nuclear factor-*κ*B/p65. *Clinical and Experimental Pharmacology and Physiology*.

[22] Mahomoodally M. F. (2013). Traditional medicines in Africa: an appraisal of ten potent African medicinal plants. *Evidence-Based Complementary and Alternative Medicine*.

[23] Mehrabani D., Ziaei M., Hosseini S. V. (2011). The effect of *Calendula Officinalis* in therapy of acetic acid induced ulcerative colitis in dog as an animal model. *Iranian Red Crescent Medical Journal*.

[24] Tanideh N., Nematollahi S. L., Hosseini S. V. (2014). The healing effect of *Hypericum perforatum* extract on acetic acid-induced ulcerative colitis in rat. *Annals of Colorectal Research*.

[25] Rajendiran V., Natarajan V., Devaraj S. N. (2018). Anti-inflammatory activity of Alpinia officinarum Hance on rat colon inflammation and tissue damage in DSS induced acute and chronic colitis models. *Food Science and Human Wellness*.

[26] Shin E. J., Sung M. J., Yang H. J., Kim M. S., Hwang J. T. (2014). *Boehmeria nivea* attenuates the development of dextran sulfate sodium-induced experimental colitis. *Mediators of Inflammation*.

[27] Moronkola D. O., Kunle O. F. (2012). Essential oil compositions of leaf, stems bark and root of *Alstonia boonei* (De Wild Apocynaceae). *International Journal of Biological Pharmaceutical Research*.

[28] Adotey J. P. K., Adukpo G. E., Boahen Y. O., Armah F. A. (2012). A review of the ethnobotany and pharmacological importance of *Alstonia boonei* De wild (Apocynaceae). *ISRN Pharmacology*.

[29] Kweifo-Okai G., Bird D., Field B. (1995). Antiinflammatory activity of a Ghanaian antiarthritic herbal preparation: III. *Journal of Ethnopharmacology*.

[30] Akinmoladun A. C., Ibukun E. O., Afor E. (2007). Chemical constituents and antioxidant activity of *Alstonia boonei*. *African Journal of Biotechnology*.

[31] Opoku F., Akoto O. (2015). Antimicrobial and Phytochemical Properties of Alstonia Boonei Extracts. *Organic Chemistry: Current Research*.

[32] Olajide O. A., Awe S. O., Makinde J. M. (2000). Studies on the anti-inflammatory, antipyretic and analgesic properties of Alstonia boonei stem bark. *Journal of Ethnopharmacology*.

[33] Odugbemi T. O., Akinsulire O. R. (2008). Medicinal plants useful for malaria therapy in Okeigbo, Ondo State, Southwest, Nigeria. *African Journal of Traditional, Complementary and Alternative Medicines*.

[34] Chime S. A., Ugwuoke E. C., Onyishi I. V., Brown S. A., Onunkwo G. C. (2013). Formulation and evaluation of *Alstonia boone*i stem bark powder tablets. *Indian Journal of Pharmaceutical Sciences*.

[35] Okoye N. N., Ajaghaku D. L., Okeke H. N., Ilodigwe E. E., Nworu C. S., Okoye F. B. C. (2014). beta-Amyrin and alpha-amyrin acetate isolated from the stem bark of Alstonia boonei display profound anti-inflammatory activity. *Pharmaceutical Biology*.

[36] Obiagwu M. O., Ihekwereme C. P., Ajaghaku D. L., Okoye F. B. C. (2014). The useful medicinal properties of the root-bark extract of Alstonia boonei (apocynaceae) may be connected to antioxidant activity. *ISRN Pharmacology*.

[37] Enechi O. C., Oluka I. H., Ugwu O. P. C. (2014). Acute toxicity, lipid peroxidation and ameliorative properties of *Alstonia boonei* ethanol leaf extract on the kidney markers of alloxan induced diabetic rats. *African Journal of Biotechnology*.

[38] Nkono Ya Nkono B. L., Dongmo Sokeng S., Dzeufiet Djomeni P. D., Longo F., Kamtchouing P. (2015). Subchronic toxicity of aqueous extract of *Alstonia boonei* de wild. (apocynaceae) stem bark in normal rats. *International Journal of Pharmacology and Toxicology*.

[39] Matos F. J. A. (1997). *Introducao à Fitoqu´Imica Experimental*.

[40] Mesaik M., Zaheerulhaq Z., Murad S. (2006). Biological and molecular docking studies on coagulin-H: human IL-2 novel natural inhibitor. *Molecular Immunology*.

[41] Mahomoodally M. F., Gurib-Fakim A., Subratty A. H. (2007). Effect of exogenous ATP on *Momordica charantia* Linn. (Cucurbitaceae) induced inhibition of D-glucose, L-tyrosine and fluid transport across rat everted intestinal sacs in vitro. *Journal of Ethnopharmacology*.

[42] Mbiantcha M., Almas J., Shabana S. U., Nida D., Aisha F. (2017). Anti-arthritic property of crude extracts of Piptadeniastrum africanum (Mimosaceae) in complete Freund’s adjuvant-induced arthritis in rats. *BMC Complementary and Alternative Medicine*.

[43] Azadmehr A., Maliji G., Hajiaghaee R., Shahnazi M., Afaghi A. (2012). Inhibition of pro-inflammatory cytokines by ethyl acetate extract of *Scrophularia striata*. *Tropical Journal of Pharmaceutical Research*.

[44] Philippe L., Gegout-Pottie P., Guingamp C. (1997). Relations between functional, inflammatory, and degenerative parameters during adjuvant arthritis in rats. *American Journal of Physiology*.

[45] Heaton K. W., Lewis S. J. (2007). Stool form scale as a useful guide to intestinal transit time. *Scandinavian Journal of Gastroenterology*.

[46] Kaser A., Zeissig S., Blumberg R. S. (2010). Inflammatory bowel disease. *Annual Review of Immunology*.

[47] D’Argenio G., Mazzone G., Tuccillo C. (2012). Apple polyphenols extract (APE) improves colon damage in a rat model of colitis. *Digestive and Liver Disease*.

[48] Strober W., Fuss I., Mannon P. (2007). The fundamental basis of inflammatory bowel disease. *Journal of Clinical Investigation*.

[49] Okayasu I., Hatakeyama S., Yamada M., Ohkusa T., Inagaki Y., Nakaya R. (1990). A novel method in the induction of reliable experimental acute and chronic ulcerative colitis in mice. *Gastroenterology*.

[50] Tanaka T., Kohno H., Suzuki R., Yamada Y., Sugie S., Mori H. (2003). A novel inflammation-related mouse colon carcinogenesis model induced by azoxymethane and dextran sodium sulfate. *Cancer Science*.

[51] Cooper H. S., Murthy S. N., Shah R. S., Sedergran D. J. (1993). Clinicopathologic study of dextran sulfate sodium experimental murine colitis. *Laboratory Investigation*.

[52] Ghia J. E., Blennerhassett P., Collins S. M. (2008). Impaired parasympathetic function increases susceptibility to inflammatory bowel disease in a mouse model of depression. *Journal of Clinical Investigation*.

[53] Wallace J. L. (2006). COX-2: a pivotal enzyme in mucosal protection and resolution of inflammation. *The Scientific World Journal*.

[54] Hess A., Axmann R., Rech J. (2011). Blockade of TNF-*α* rapidly inhibits pain responses in the central nervous system. *Proceedings of the National Academy of Sciences the United States of America*.

[55] Bento A. F., Claudino R. F., Dutra R. C., Marcon R., Calixto J. B. (2011). Omega-3 fatty acid derived mediators 17 (R)-hydroxy docosahexaenoic acid, aspirin-triggered resolvin D1 and resolvin D2 prevent experimental colitis in mice. *The Journal of Immunology*.

[56] Danese S., Semeraro S., Marini M. (2005). Adhesion molecules in inflammatory bowel disease: therapeutic implications for gut inflammation. *Digestive and Liver Disease*.

[57] Dieleman L. A., Ridwan B. U., Tennyson G. S. (1994). Dextran sulfate sodium-induced colitis occurs in severe combined immunodeficient mice. *Gastroenterology*.

[58] Chami B., Yeung A. W., van Vreden C., King N. J. C., Bao S. (2014). The role of CXCR3 in DSS-induced colitis. *PLoS One*.

[59] Kiesler P., Fuss I. J., Strober W. (2015). Experimental models of inflammatory bowel diseases. *Cellular and Molecular Gastroenterology and Hepatology*.

[60] Lima E. M., Nascimento A. M., Lenz D. (2014). Triterpenes from the Protium heptaphyllum resin—chemical composition and cytotoxicity. *Revista Brasileira de Farmacognosia*.

[61] Silva J. R. L., da Silva M. D. P., Lefort J., Vargaftig B. B. (2000). Endotoxins, asthma, and allergic immune responses. *Toxicology*.

[62] Harris S. G., Padilla J., Koumas L., Ray D., Phipps R. P. (2002). Prostaglandins as modulators of immunity. *Trends in Immunology*.

[63] Almas J. (2013). Study of the suppression of inflammatory arthritis at molecular level by natural and synthetic inhibition of TNF*α* and IL-1*β*.

[64] Rabi T., Shukla S., Gupta S. (2008). Betulinic acid suppresses constitutive and TNF*α*—induced NF—*κ*B activation and induces apoptosis in human prostate carcinoma PC—3 cells. *Molecular Carcinogenesis*.

[65] Yu-Jin H., Jaewhan S., Haeng-Ran K., Kyung A. H. (2014). Oleanolic acid regulates NF-*κ*B signaling by suppressing MafK expression in RAW 264.7 cells. *BMB Reports*.

[66] Vitor C., Figueiredo C., Hara D., Bento A., Mazzuco T., Calixto J. (2009). Therapeutic action and underlying mechanisms of a combination of two pentacyclic triterpenes, *α*- and *β*-amyrin, in a mouse model of colitis. *British Journal of Pharmacology*.

[67] Beserra F. P., Xue M., Maia G. L. A. (2018). Lupeol, a Pentacyclic Triterpene, Promotes migration, wound closure, and contractile effect *in vitro*: possible involvement of PI3K/akt and p38/ERK/MAPK pathways. *Molecules*.

[68] Weyermann J., Lochmann D., Zimmer A. (2005). A practical note on the use of cytotoxicity assays. *International Journal of Pharmaceutics*.

[69] Lu F. C. (1992). General data, evaluation procedures, target organs and assessment of risk. *Toxicology Masson Paris*.

[70] Carrier J. C., Aghdassi E., Jeejeebhoy K., Allard J. P. (2006). Exacerbation of dextran sulfate sodium-induced colitis by dietary iron supplementation: role of NF-*κ*B. *International Journal of Colorectal Disease*.

[71] Kretzmann N. A., Fillmann H., Mauriz J. L. (2008). Effects of glutamine on proinflammatory gene expression and activation of nuclear factor kappa B and signal transducers and activators of transcription in TNBS-induced colitis. *Inflammatory Bowel Diseases*.

[72] Coburn L. A., Gong X., Singh K., Asim M., Scull B. P., Allaman M. M. (2012). L-Arginine supplementation improves responses to injury and inflammation in dextran sulfate sodium colitis. *PLoS ONE*.

[73] Moura R. M., Hartmann R. M., Licks F. (2016). Antioxidant effect of mesalazine in the experimental colitis model induced by acetic acid. *Journal of Coloproctology*.

[74] Jabri M.-A., Rtibi K., Tounsi H. (2015). Myrtle berry seed aqueous extract inhibits human neutrophil myeloperoxidase in vitro and attenuates acetic acid-induced ulcerative colitis in rats. *RSC Advances*.

[75] Ek R. O., Serter M., Ergin K. (2014). Protective effects of citicoline on TNBS-induced experimental colitis in rats. *International journal of clinicalf and Experimental medicine*.

[76] Nooh H. Z., Nour-Eldien N. M. (2016). The dual anti-inflammatory and antioxidant activities of natural honey promote cell proliferation and neural regeneration in a rat model of colitis. *Acta Histochemica*.

[77] Al-Rejaie S. S., Abuohashish H. M., Al-Enazi M. M., Al-Assaf A. H., Parmar M. Y., Ahmed M. M. (2013). Protective effect of naringenin on acetic acid-induced ulcerative colitis in rats. *World Journal of Gastroenterology*.

[78] Sathyasaikumar K. V., Swapna I., Reddy P. V. B. (2007). Fulminant hepatic failure in rats induces oxidative stress differentially in cerebral cortex, cerebellum and pons medulla. *Neurochemical Research*.

[79] Hartmann R. M., Morgan Martins M. I., Tieppo J., Fillmann H. S., Marroni N. P. (2012). Effect of *Boswellia serrata* on antioxidant status inan experimental model of colitis rats induced by acetic acid. *Digestive Diseases and Sciences*.

[80] Alvarez-Suarez J. M., Giampieri F., Cordero M. (2016). Activation of AMPK/Nrf2 signalling by Manuka honey protects human dermal fibroblasts against oxidative damage by improving antioxidant response and mitochondrial function promoting wound healing. *Journal of Functional Foods*.

[81] Kongkachuichai R., Charoensiri R., Sungpuag P. (2010). Carotenoid, flavonoid profiles and dietary fiber contents of fruits commonly consumed in Thailand. *International Journal of Food Sciences and Nutrition*.

[82] Rabbani G. H., Teka T., Zaman B., Majid N., Khatun M., Fuchs G. J. (2001). Clinical studies in persistent diarrhea: dietary management with green banana or pectin in Bangladeshi children. *Gastroenterology*.

[83] Adjouzem C. F. (2016). Propriétés antidiarrhéique et antibactérienne des extraits aqueux et méthanolique des écorces du tronc de Alstonia boonei (Apocynaceae) chez le rat.

